# Supplementary material for: Tight hydrophobic core and flexible helices yield MscL with a high tension gating threshold and a membrane area mechanical strain buffer
Source: Front Chem. 2023 May 24;11:1159032. doi: 10.3389/fchem.2023.1159032 (PMC10244533; doi:10.3389/fchem.2023.1159032)
Supplement: Supplementary file 1 [file DataSheet1.pdf]

Supplementary Material

Tight hydrophobic core and flexible helices yield MscL with a high tension gating threshold and a membrane area mechanical strain buffer

Arjun Sharma[1]**, Andriy Anishkin**[2]**, Sergei Sukharev**[2]**, and Juan M. Vanegas**[1,3,*]
[1]*Department of Physics, University of Vermont, Burlington, Vermont, USA*
[2]*Department of Biology, University of Maryland, College Park, Maryland, USA*
[3]*Current address: Department of Biochemistry and Biophysics, Oregon State University, Corvallis, Oregon, USA*

Correspondence*: Juan M. Vanegas, vanegasj@oregonstate.edu

1 SUPPLEMENTARY FIGURES

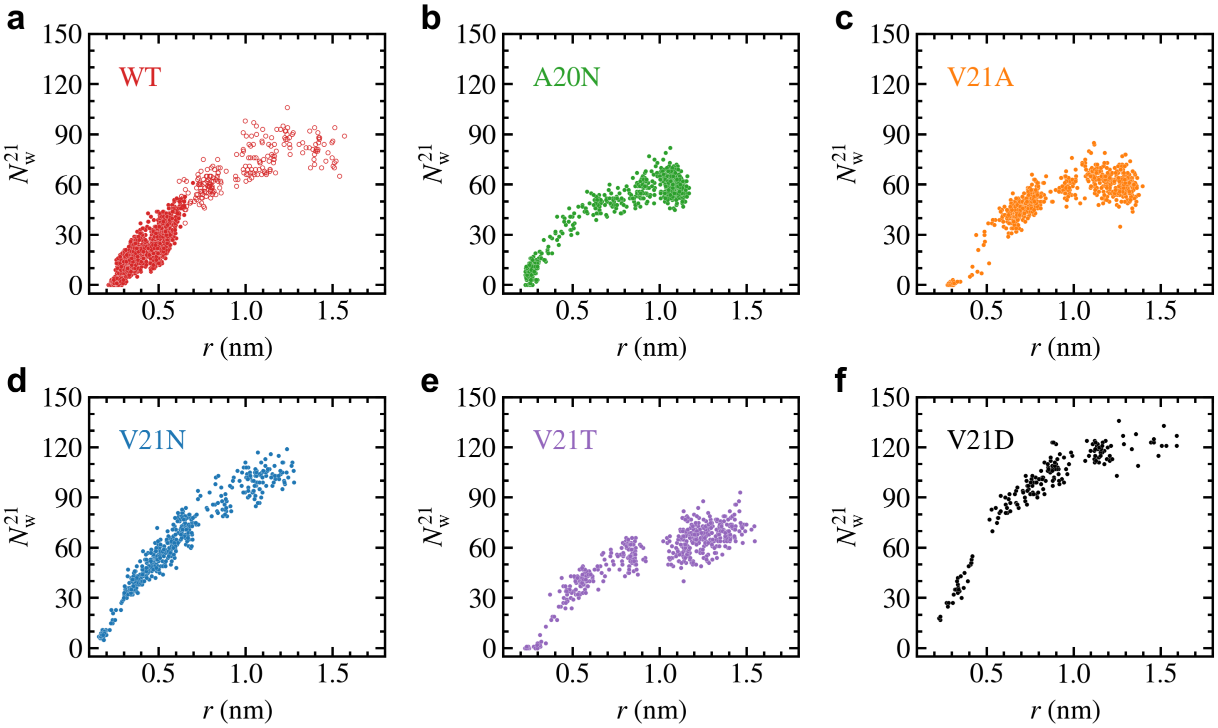

Figure S1. Hydration of the inner pore measured by the number of water molecules within 0.5 nm of residue 21, N_W^{21}, as a function of the pore radius also measured at residue 21, r.

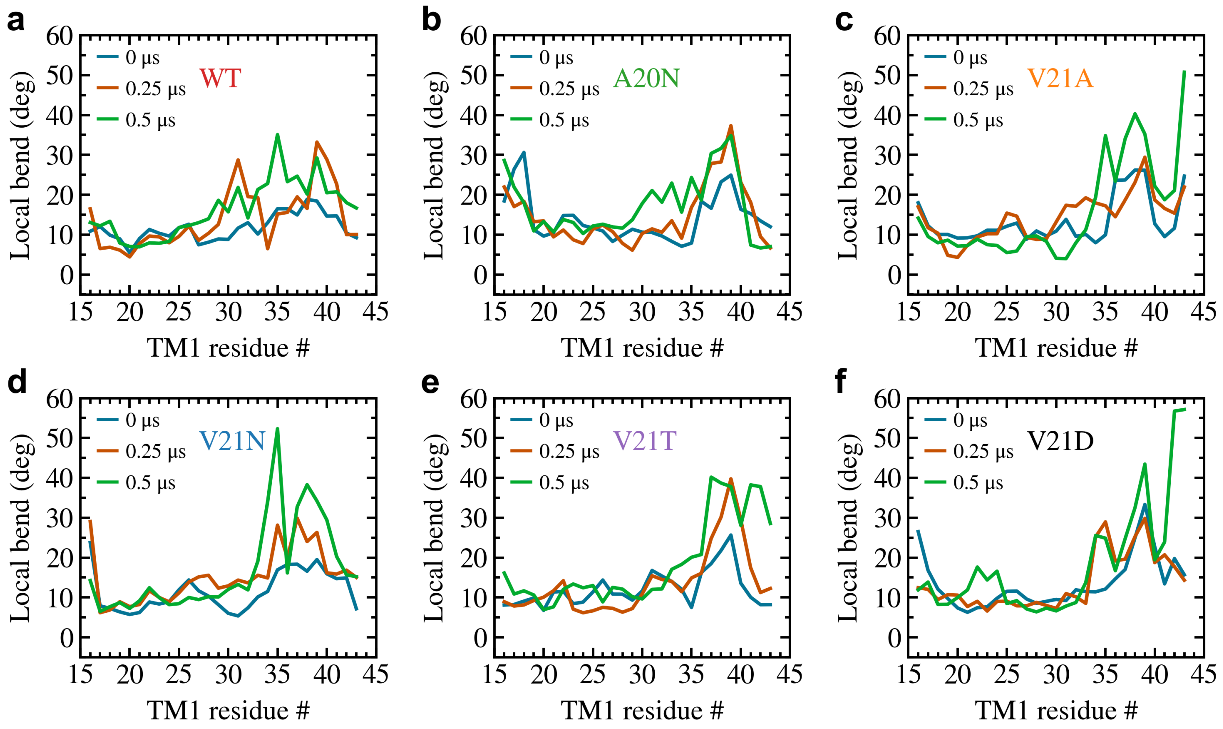

Figure S2. Local helix bend angles along TM1 (residues 13-46) as measured by the vectors of neighboring local helix axes, defined by 3 contiguous C_α carbons, according to the HELANAL algorithm (see Methods in the main text). Different colors show data for systems under high tension at $t = 0$ (blue), $t = 0.25$ (red), and $t = 0.5$ μs (green).

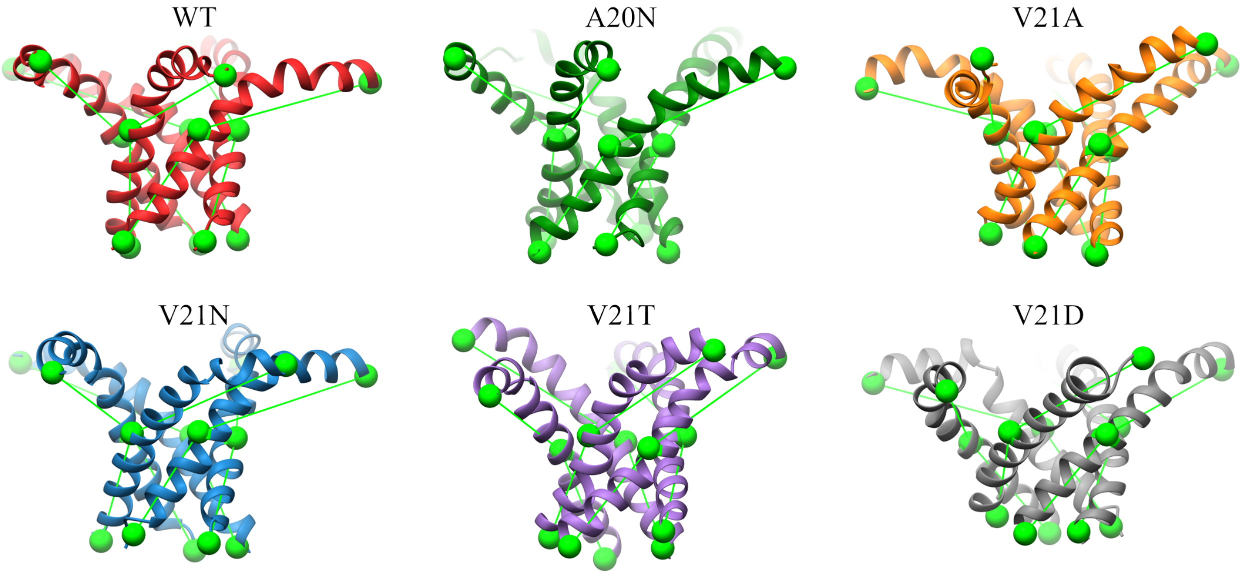

Figure S3. Ribbon representations of TM1 helices (residues 13-46) of WT and GOF mutants under high tension at $t = 0.25$ μs. Bright green spheres show the C_α atoms of residues N13, A26, and I46 which are used to define the subunit-averaged TM1 bend angle, $\langle \theta_{1,\mathrm{M}} \rangle$. All other protein elements and other membrane/solvent atoms not shown for clarity.
